# Book Notices

**Published:** 1881-11-20

**Authors:** 


### *BOOK NOTICES.*

GENERAL MEDICAL CHEMISTRY, for the use of Practitioners of Medicine. By R. A. Witthaus, A. M. M. D., Prof. of Chemistry and Toxocology, in the Medical Department of the University of the city of New York; Member of the Chemical Societies of Paris and Berlin; Fellow of the New York Academy of Medicine, and the American Academy of Medicine, etc., New York. Wm. Wood & Co. McCarty & Laird, Agents, 17 East Alabama Street, Atlanta, Georgia.

This is a work of 443 oc. pages. It is condensed and practical, yet sufficiently full for the practitioner, inasmuch as the "Bearings of Chemistry upon physiology, hygiene, therapeutics and toxicology" are given.

The modern system of notation has been followed. The metric system in weights and measures, and the centigrade scales in temperature is used throughout the work.

A MANUAL OF HISTOLOGY. Edited and prepared by Thos. S. Sallerthwaitt, M. D., of New York, President of the New York Pathological Society, Pathologist to the St. Luke's and Presbyterian Hospital, etc. In association with Drs. Tho. Dwight, J. C. Warren, Wm. F. Whitney, Chas. J. Blake, and C. H. Williams, of Boston; Dr. J. H. Sims, of Philadelphia; Dr. B. F. Westbrook, of Brooklyn, and Drs. Edmund C. Wendt, Abraham Mayer, R. W. Amidon, A. R. Robinson, W. R. Beysall, D. B. Delevan, C. L. Dana, and W. H. Porter, of New York City. With one hundred and ninety-eight illustrations. William Wood & Co. McCarty & Laird, Agents, Atlanta, Ga.

This is a large oc. 478 pages, neatly bound and beautifully illustrated. The work is timely and appropriate. It is the very thing needed. The author truly remarks that "the present time seemed opportune for its appearance, since we have latterly made much positive advance in histological studies, while histologists themselves are now more of one mind in microscopical matters. That such a book should appear under American auspices seemed further to be eminently proper, as we have in various parts of the country a goodly number of medical men who are either engaged in teaching histology or studying some special branch of it."

INDIGESTION, BILIOUSNESS AND GOUT IN ITS PROTEAN ASPECTS. By J. Milner Fothergill, M. D., Member of the Royal College of Physicians of London; Senior Assistant Physician to the City of London Hospital for diseases of the chest (Victoria

Park); late Assistant Physician to the West London Hospital; Associate Fellow of the College of Physicians of Philadelphia. New York, Wm. Wood & Co., 1881. McCarty & Laird, Agents, Atlanta, Ga.

The above is a neat work of 316 oc. pages. It contains many useful and practical suggestions upon a highly important subject. We were most favorably impressed with the parts of the work relating to diet. The practicing physician will find it very interesting and useful to his library.

THE PRESCRIBERS' MEMORANDA. New York, William Wood & Co., 1881.

A little work of 301 duod. pages. We find no author's name attached to the work, yet we are constrained to say that it is an excellent book, containing numerous admirable formulæ adapted to nearly every phase of disease.

THE WILDERNESS CURE, by Marc Cook, author of "Camp Lore." New York, William Wood & Co., pp 253. 1881. McCarty & Laird, agents, Atlanta, Ga.

This work is valuable, and may well be read by the practitioner. The great benefits of the climatic treatmen tof disease, especially in lung affections, will here be found practicaliy illustrated. No system or plan ever yet devised promised so much for the consumptive as outdoor diversion and camp-life. This work tells how best to manage to secure its full benefits.

CHEMICAL ANALYSES OF THE URINE: Based, in part, on Casselmann's Analyse Des Urines. By Edgar F. Smith, Ph. D., Prof. of Chemistry in Muhlenburg College, and John Marshall, M. D., Dean, of Chem. Med. Depart. University of Penn.; with illustrations. Philadelphia: Pressley Blakiston, 1881. S. P. Richards, Atlanta, Ga. Price, $1.00.

This work is highly instructive and satisfactory to the student and practitioner desiring to learn the best methods for chemical analyses of the urine.

TRANSACTIONS OF THE AMERICAN GYNECOLOGICAL SOCIETY. Vol. five, for year 1881. Boston: Houghton, Mifflin & Co. Octavo pages 470.

A very neat and creditable volume, and exceedingly interesting. It "contains the index to the Gynecological and Obstetrical Literature of all countries, for the year 1879, prepared with cooperation of Dr. J. S. Billings, U. S. A., in charge of the National Medical Library in Washington."

The work contains a large number of original papers, some of which are of rare ability and interest.

